# Gut Microbial Colonization Orchestrates TLR2 Expression, Signaling and Epithelial Proliferation in the Small Intestinal Mucosa

**DOI:** 10.1371/journal.pone.0113080

**Published:** 2014-11-14

**Authors:** Nives Hörmann, Inês Brandão, Sven Jäckel, Nelli Ens, Maren Lillich, Ulrich Walter, Christoph Reinhardt

**Affiliations:** Center for Thrombosis and Hemostasis (CTH), University Medical Center Mainz, Junior Group Translational Research in Thrombosis and Hemostasis, Mainz, Germany; IRCCS Istituto Oncologico Giovanni Paolo II, Italy

## Abstract

The gut microbiota is an environmental factor that determines renewal of the intestinal epithelium and remodeling of the intestinal mucosa. At present, it is not resolved if components of the gut microbiota can augment innate immune sensing in the intestinal epithelium via the up-regulation of Toll-like receptors (TLRs). Here, we report that colonization of germ-free (GF) Swiss Webster mice with a complex gut microbiota augments expression of TLR2. The microbiota-dependent up-regulation of components of the TLR2 signaling complex could be reversed by a 7 day broad-spectrum antibiotic treatment. TLR2 downstream signaling via the mitogen-activated protein kinase (ERK1/2) and protein-kinase B (AKT) induced by bacterial TLR2 agonists resulted in increased proliferation of the small intestinal epithelial cell line MODE-K. Mice that were colonized from birth with a normal gut microbiota (conventionally-raised; CONV-R) showed signs of increased small intestinal renewal and apoptosis compared with GF controls as indicated by elevated mRNA levels of the proliferation markers Ki67 and Cyclin D1, elevated transcripts of the apoptosis marker Caspase-3 and increased numbers of TUNEL-positive cells per intestinal villus structure. In accordance, TLR2-deficient mice showed reduced proliferation and reduced apoptosis. Our findings suggest that a tuned proliferation response of epithelial cells following microbial colonization could aid to protect the host from its microbial colonizers and increase intestinal surface area.

## Introduction

The intestine shows intense self-renewal kinetics originating from the stem cell niche situated in the crypts of Lieberkühn [1] with the mucus layer on its epithelial surface being habitat to trillions of microbes [2]. The gut microbiota is an environmental factor that strongly impacts on morphology and cell renewal in the small intestine [3]. Microbiota-dependent effects on cellular proliferation and intestinal tissue homeostasis can best be studied by the use of germ-free (GF) mouse technology and colonization experiments with a complex gut microbiota or even by colonization with defined microbial strains. Colonization of GF mice with a complex gut microbiota induces tissue remodeling and the architecture of the small intestine is rigorously changed as small intestinal villi are shortened and widened [4], [5]. In addition to angiopoietin-1 dependent vascularization of the distal small intestinal mucosa, which we have shown to depend on protease-activated receptor 1 (PAR1) [5] colonization with a gut microbiota also evokes massive proliferation of the epithelial lineage [3], [6] that forms a barrier between gut resident microbes and the underlying lamina propria [7].

It has been reported by several groups that epithelial cell turn over is about twice as fast in the small intestine of colonized mice compared with GF controls [3], [6]. However, the exact signaling pathways that sense gut microbial signatures and trigger expansion of the small intestinal epithelial lining have not been unequivocally dissected. A recent study that was based on murine crypt organoid cultures has implicated the pattern recognition receptor nucleotide-binding oligomerization domain-containing protein-2 (Nod2) in renewal of the intestinal epithelium from the intestinal stem cell niche triggered by the peptidoglycan motif muramyl dipeptide [8]. While renewal of the epithelial lineage from stem cells has been defined [1], [8], the induction of proliferative signaling in differentiated polarized intestinal epithelial cells has gained less attention. In addition to cytosolic Nod receptors, TLRs represent major sensors of the host that mediate a magnitude of cellular responses to microbial challenges [7]. TLR signaling pathways are either classified as dependent on the adaptor molecule myeloid differentiation primary response gene 88 (MyD88); e.g. TLR1, TLR2 and TLR6 or as TIR-domain-containing adapter-inducing interferon-β (TRIF)-dependent pathways, e.g. TLR3 and TLR4 [7], [9]. TLRs contain an ectodomain consisting of leucin-rich repeats that is responsible for ligand binding. Upon dimerization the Toll/interleukin-1 receptor homologous region (TIR-domain) interacts with the adaptor molecules MyD88, MAL/TIRAP (toll/interleukin-1-receptor-domain-containing adaptor protein), TRAM (TRIF-related adaptor molecule) and TRIF resulting in autophosphorylation of IL-1 receptor associated kinase (IRAK). Ultimately, this leads to increased phosphorylation of ERK1/2 (MAP-kinase), to AKT signaling and to activation of the transcription factor NF-κB [7]. In spite of the constant exposure to luminal bacteria, the intestinal epithelium tolerates the presence of luminal pathogen-associated molecular patterns (PAMPs) and can avoid the development of acute inflammatory immune responses via several mechanisms [10], e.g. spatial differences in expression of TLR signaling complexes and altered cellular distribution [11]–[13], down-regulation of pattern recognition receptors (TLRs) [14] and blockage in the post-receptor signaling cascade via TOLLIP (Toll interacting protein) [15], [16]. Various studies have reported expression of TLR1, TLR2, TLR3, TLR4, TLR5 and TLR9 in small intestinal epithelial cells [7]. In mucosal scrapings of the colon expression levels of TLR2 and TLR5, but not TLR1, 3, 4, 6 and 9 were found regulated by the gut microbiota [14]. At present, it is unclear whether expression of TLR2 signaling complexes in the small intestine can also be regulated by colonization with gut microbial communities. Moreover, a possible contribution of TLR2 signaling to epithelial cell renewal is unexplored. Here, we show by taking advantage of GF mouse models that the gut microbiota regulates TLR signaling in the intestinal epithelium and we demonstrate that induction of the TLR2 pathway impacts on proliferation of small intestinal epithelial cells.

## Methods

### Animals

All mice were housed and experiments were performed according to the German Animal Welfare Act in a barrier facility (ZVTE, Zentrale Versuchstiereinrichtung, Universitätsmedizin Mainz) with a 12-hour light-dark cycle. Mice were kept in EU Type II IVC cages with at maximum 5 mice per cage under specific pathogen-free (SPF) conditions. Germ-free (GF) Swiss Webster or C57BL/6 mice were maintained in sterile flexible film isolators. The GF status of the animals was verified every second week by anaerobic culturing and 16S bacterial DNA PCR with universal primers. GF and CONV-R Swiss Webster mice were fed an autoclaved chow diet (LabDiet, St. Louis, MI) and drinking water *ad libitum*. SPF C57BL/6J (WT), Tlr2^−/−^ and Tlr5^−/−^ mice were originally purchased from The Jackson Laboratory (Bar Harbor, ME). SPF Tlr4^−/−^, Myd88^−/−^ and Trif^−/−^ animals were kindly provided by Markus P. Radsak (Institute of Immunology, Johannes Gutenberg University, Mainz, Germany). SPF Tlr7^−/−^ mice were received by Kerstin Steinbrink (Dermatology Clinics, University Medical Center Mainz, Germany). Sex matched mice 10–14 weeks of age were used. GF Swiss Webster mice were colonized for 14 days with a complex gut microbiota harvested from the cecum of a CONV-R donor mouse. GF C57BL/6 mice were monocolonized with *Escherichia coli* strain JP 313 (kindly provided by Evelyne Turlin, Institute Pasteur, Paris) in germ-free flexible film isolators. For decimation of colonizing gut microbes CONV-R Swiss Webster mice were treated with broad-spectrum antibiotics (1 g/L ampicillin, Sigma-Aldrich, St. Louis, MO and 0.5 g/L neomycin, Sigma-Aldrich) for 7 days via the drinking water according to an established protocol [17]. Antibiotic decimation of gut bacteria was analyzed by qPCR with universal 16S primers [18]. Treated mice were under daily surveillance. Mice were sacrificed by cervical dislocation. All procedures performed on mice were approved by the Institutional Animal Care and Use Committee (IACUC; Landesuntersuchungsamt Rheinland-Pfalz, Koblenz, Germany; G11–1–025).

### Cell culture

MODE-K cells were kindly provided by Dominique Kaiserlian (INSERM, Cedex, France) and maintained as described [19]. Cells were seeded in 6-well plates and stimulated at confluency. Prior to stimulation cells were washed once with PBS pH 7.2 and medium containing 10% FCS with or without PAMPs (Lipopolysaccharide from *Escherichia coli* 0111:B4 (Sigma-Aldrich, St. Louis, MO) –100 ng/ml; Peptidoglycan from *Bacillus subtilis* (Sigma-Aldrich, St. Louis, MO) –50 µg/ml; Lipoteichoic acid from *Streptococcus faecalis* (Sigma-Aldrich, St. Louis, MO) –10 µg/ml; Macrophage-activating Lipopeptide-2 (Alexis Biochemicals, San Diego, CA) –2 µg/ml; Heat-killed *Listeria monocytogenes* (Invivo Gen, San Diego, CA) –2×10^8^; Pam3CSK4 (Invivo Gen. San Diego, CA) –0.5 µg/ml was added for the times indicated. Primary enterocytes from mice were isolated [5]. Mice were sacrificed and the small intestine was excised, content was removed by repeated washing with PBS (pH 7.2, w/o Ca^2+^/Mg^2+^). Specimens were opened longitudinally and dissected in pieces of 5 mm. After digestion in cell recovery solution (BD Bioscience, San Jose, CA) for 60 min on ice, enterocytes were scraped off with an inoculating loop. Cells were washed twice in 50 ml PBS (pH 7.2, w/o Ca^2+^/Mg^2+^), centrifuged at 1200 g for 4 min and subsequently washed twice with 50 ml PBS (pH 7.2, with Ca^2+^/Mg^2+^). Cells from one specimen were plated on one 6-well plate in basal medium (D-MEM, 4,5 g/l glucose, with 1% sodium pyruvate, 1% Pen/Strep) for 4 hours. Medium was removed and adhering cells were harvested in cell lysis buffer (50 mM Tris-HCl pH 8.0, 150 mM sodium chloride, 5 mM EDTA, 1% Triton X-100) containing Roche complete protease inhibitor cocktail (Roche, Penzberg, Germany). Knockdown experiments were performed by transfection of 1 µg of 4 pooled siRNAs against TLR2 (Thermo Scientific, Waltham, MA) or scrambled control siRNA. For transfection Dharmafect 4 (Thermo Scientific, Waltham, MA) was used as described in the protocol provided. For proliferation analyses, cells were seeded in 96-well plates, transfected with siRNA and subsequently used for proliferation measurements. BrdU solution (Becton Dickinson, Franklin Lakes, NJ) was added overnight. For proliferation measurements of TLR-agonist treated cells, wells were seeded in 96 well plates for 2 days. After stimulation for 2 to 8 hours BrdU solution was added and the ELISA-based assay was performed according to the manufacturer's protocol (BrdU Proliferation Kit, Cell Signaling, Danvers, MA).

### Preparation of intestinal samples

For mRNA and Western blot analysis, segments were frozen in liquid nitrogen directly after dissection. For immunoblots, the flash-frozen fifth segment corresponding to ileum was homogenized in cell lysis buffer (50 mM Tris-HCl pH 8.0, 150 mM sodium chloride, 5 mM EDTA, 1% Triton X-100) containing Roche Complete protease and PhosStop phosphatase inhibitors (Roche, Penzberg, Germany). The homogenate was incubated for 30 min on ice and centrifuged three times at 9000 g for 10 min to remove insoluble cell debris. Protein amounts were measured using the DC Protein Assay (Bio-Rad Laboratories, Berkeley, CA).

### Histological analyses

Tissue specimens were harvested as described. The small intestine was rinsed with PBS to remove content. Tissue was fixed over night at 4°C in formalin solution (4% formaldehyde in PBS, pH 7.4) and embedded in paraffin. Caspase-3 stainings were performed using the rabbit-anti-Caspase 3 antibody (Asp175, 1∶250 dilution, Cell Signaling, Danvers, MA) recognizing cleaved Caspase-3 by the Core Facility for Histology (University Medical Center Mainz) according to established protocols. TUNEL staining was performed using the *in situ* cell death detection kit, fluorescein (Roche, Penzberg, Germany) according to manufacturer's protocol.

### qRT–PCR analysis

Total RNA was isolated from small intestinal tissues and cultivated cells with the RNeasy Kit (Qiagen, Hilden, Germany). The small intestine was divided into 8 equal segments and segment 5 corresponding to ileum was analyzed. On-column digestion of genomic DNA was performed according to manufacturer's protocol. Total RNA (2 µg) was reverse transcribed (High Capacity cDNA Reverse Transcription Kit; Applied Biosystems, Foster City) and SYBR green-based qRT–PCR was performed with iQ SYBR Green Supermix (Bio Rad Laboratories, Berkeley, CA) with oligonucleotides specified in Table 1.

**Table 1:** Primer Table.

Primer Abbreviation	Recognized cDNA	Oligonucleotide Sequence
mL32_for	60S ribosomal protein L32	TGGCTCCTTCGTTGCTGCTG
mL32_rev	60S ribosomal protein L32	CTGGACGGCTAATGCTGGTG
mCasp3_for	murine Caspase 3	TGGTGATGAAGGGGTCATTTATG
mCasp3_rev	murine Caspase 3	TTCGGCTTTCCAGTCAGACTC
mTLR1_for	murine Toll-like receptor 1	TCAAGCATTTGGACCTCTCCT
mTLR1_rev	murine Toll-like receptor 1	TTCTTTGCATATAGGCAGGGC
mTLR2_for	murine Toll-like receptor 2	ACAATAGAGGGAGACGCCTTT
mTLR2_rev	murine Toll-like receptor 2	AGTGTCTGGTAAGGATTTCCCAT
mTLR4_for	murine Toll-like receptor 4	ATGGCATGGCTTACACCACC
mTLR4_rev	murine Toll-like receptor 4	GAGGCCATTTTTGTCTCCACA
mTLR6_for	murine Toll-like receptor 6	TGAGCCAAGACAGAAAACCCA
mTLR6_rev	murine Toll-like receptor 6	GGGACATGAGTAAGGTTCCTGTT
mKi67_for	murine antigen identified by monoclonal antibody Ki-67	CAATGTGCCTCGCAGTAAGA
mKi67_rev	murine antigen identified by monoclonal antibody Ki-67	GCATCTTTGGGGTTTTCTCA
mCyclinD1_for	murine Cyclin D1	GCGTACCCTGACACCAATCTC
mCyclinD2_rev	murine Cyclin D1	CTCCTCTTCGCACTTCTGCTC
mMyD88_for	murine Myeloid differentiation primary response gene (88)	AGGACAAACGCCGGAACTTTT
mMyD88_rev	murine Myeloid differentiation primary response gene (88)	GCCGATAGTCTGTCTGTTCTAGT
mTrif_for	murine TIR-domain-containing adapter-inducing interferon-β	TTGGGGACATACGTTACACTCC
mTrif_rev	murine TIR-domain-containing adapter-inducing interferon-β	CGGTGTGTTACATAGCTTGCTG
16S UniF_for	Universal primer for 16S qPCR	GTGSTGCAYGGYYGTCGTCA
16S UniR_rev	Universal primer for 16S qPCR	ACGTCRTCCMCNCCTTCCTC
8F	Universal primer for control PCRs	AGAGTTTGATCCTGGCTCAG
338R	Universal primer for control PCRs	TGCTGCCTCCCGTAGGAGT

### Immunoblotting

Tissue and cell lysates were separated by using a NuPAGE system with MOPS SDS buffer and 10% BisTris gels (Invitrogen, Carlsbad, CA). Proteins were transferred to nitrocellulose membranes (0,45 µm, GE Healthcare, Chalfont St Giles, UK). The membrane was blocked in 3% BSA (in TBS/Tween) and incubated overnight in TBS-T with 3% BSA containing the primary antibody rabbit anti-mouse TLR2 (clone EPNCIR133; dilution 1∶1000; Abcam, Cambridge, UK), rabbit anti-β-actin (A5060, dilution 1∶10.000; Sigma Aldrich, St. Louis, MO), rabbit anti-phospho-ERK 1/2, rabbit anti-ERK 1/2, rabbit anti-IκB (clone 44D4), rabbit anti-phospho-IκB antibody (clone 14D4), rabbit anti-phospho-AKT (clone S473), rabbit anti-AKT (clone C67E7) (dilution 1∶1000; Cell Signaling, Danvers, MA). Secondary goat anti-rabbit IgG (peroxidase-conjugated; Vector Labs, Burlingame, CA) was applied for 1 h. Blots were developed with enhanced chemiluminescence solution (Cell Signaling, Danvers, MA).

### Statistical Analysis

Data are expressed as mean +/− s.e.m. Statistical calculations were performed with GraphPad Prism 6 (GraphPad Software Inc, San Diego, CA) using the independent samples Student's t-test to compare two groups and Tukey post hoc test using one-way ANOVA for more than two groups. Values of P<0,05 were considered significant. *P<0.05, ** P<0.01, ***P<0.005 and **** P<0.001.

## Results

**Figure 1:**
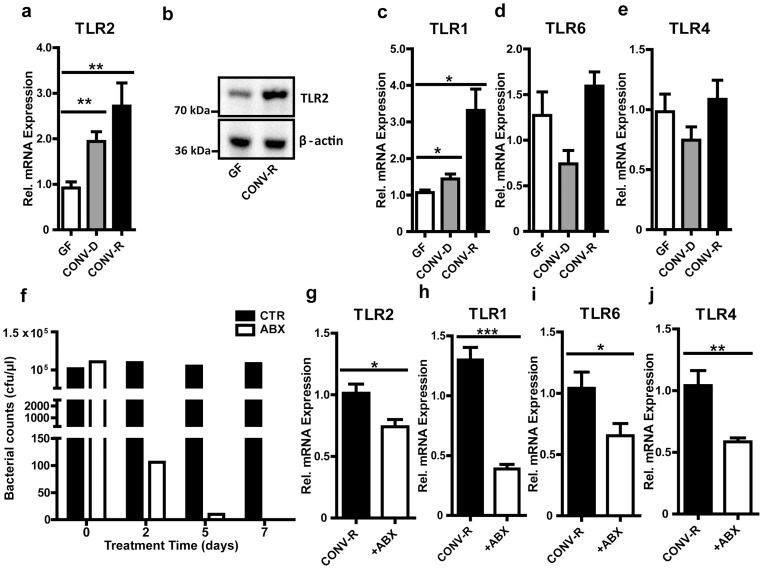
Microbial colonization leads to induction of TLR2 receptor expression in the small intestine. **a,** Relative TLR2 mRNA levels in small intestinal tissues from GF, CONV-D and CONV-R mice (n = 7 Swiss Webster mice per group). **b,** TLR2 immunoblot of isolated small-intestinal enterocyte lysates from GF and CONV-R mice (n = 6 mice per group; shown is one representative blot). **c–e,** Relative TLR1, 6 and 4 mRNA levels in small intestinal tissues from GF, CONV-D and CONV-R mice (n = 6–7 mice per group). **f,** qPCR analyses of feces samples of control mice and mice treated with antibiotics for 7 days. qPCR was performed using universal primers for 16S bacterial sequences and normed to *E. coli* bacterial counts (cfu/µl). **g–j,** Relative mRNA levels of TLR2, 1, 6 and 4 in small intestinal tissues from CONV-R mice treated with a cocktail (ABX) of ampicillin (1 g/L) and neomycin (0.5 g/L) for 7 days (n = 6–7 mice per group). Female Swiss Webster mice or cells isolated from these mice were analyzed. Results are shown as means ± s.e.m. One asterisk, P<0.05; two asterisks, P<0.01; three asterisks, P<0.005.

Since small intestinal epithelial cells express components of the TLR2 signaling complex [7], we analysed TLR2, TLR1 and TLR6 transcript levels in small intestinal tissues from GF mice compared with conventionally-raised (CONV-R) and conventional-derived (CONV-D) mice (ex-GF mice colonized for 14 days with a cecal microbiota of a CONV-R mouse). We found increased TLR2 transcripts in the small intestine both in CONV-R mice and CONV-D mice compared with GF controls (**Fig. 1a**). Importantly, in the small intestine of CONV-R mice, TLR2 was also up-regulated on the protein level (**Fig. 1b**). Furthermore, colonization with a gut microbiota resulted in increased intestinal expression of the TLR2 co-receptor TLR1 (**Fig. 1c**), but mRNA levels of the TLR2 co-receptor TLR6 were unchanged (**Fig. 1d**). In contrast to TLR2, transcript levels of TLR4 were unchanged between colonized mice and GF littermate controls (**Fig. 1e**). To investigate whether the microbiota-induced increase in TLR2 expression can be reversed by decimation of gut bacteria, we treated CONV-R mice for 7 days with a cocktail of broad-spectrum antibiotics containing ampicillin and neomycin [17]. 5 days after administration of the antibiotic cocktail a vast reduction of most gut resident bacteria was observed by 16S qPCR quantification (**Fig. 1f**) [18]. Transcripts of TLR2 and its co-receptors TLR1 and TLR6 as well as TLR4 mRNA levels were decreased if the gut microbiota was erased with antibiotics (**Fig. 1g–j**). These results suggest that the regulation of TLRs by gut microbial communities in the small intestine is a dynamic and fully reversible process.

**Figure 2:**
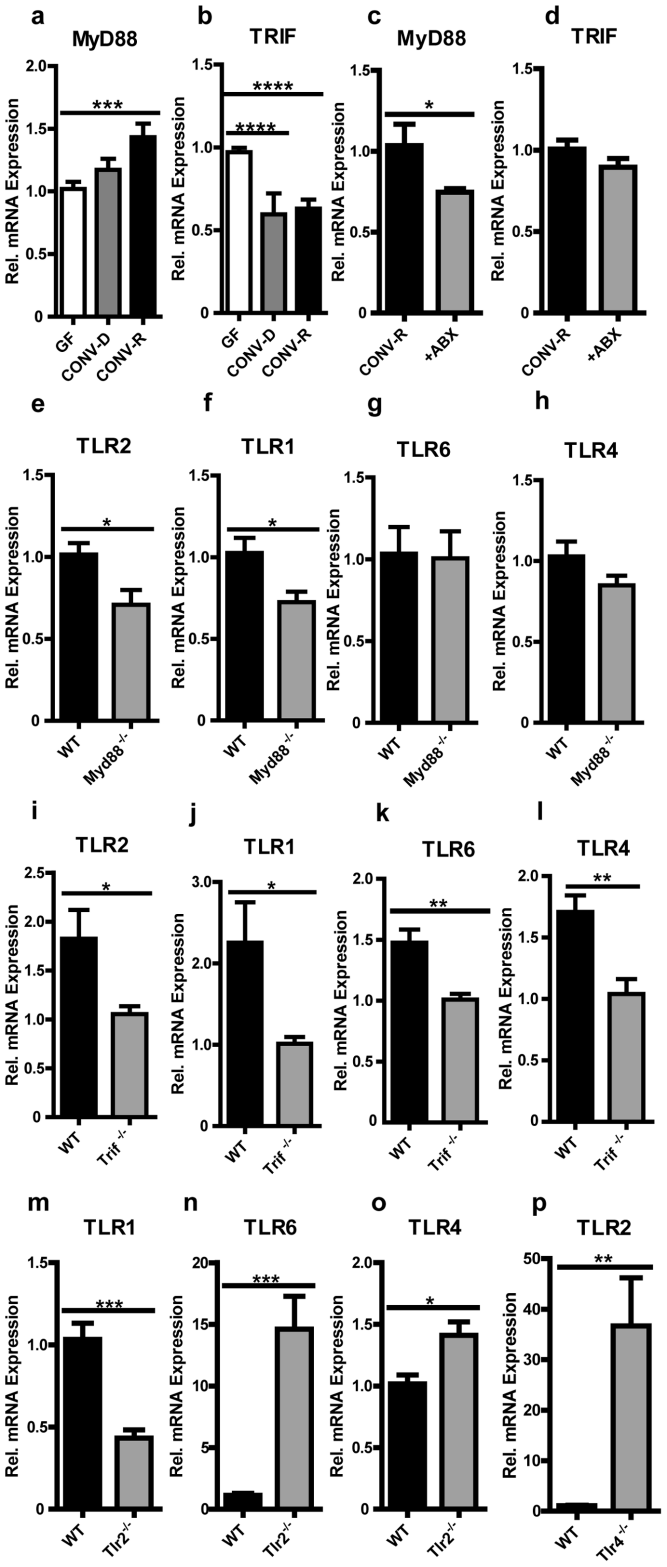
Adapter molecules MyD88 and TRIF alter expression of TLRs in the ileum – indications for TLR receptor cross-talk in the small intestine. **a+b**, Relative MyD88 and TRIF mRNA levels in small intestine from GF, CONV-D and CONV-R mice (n = 6–7 Swiss Webster mice per group). **c+d**, Relative MyD88 and TRIF mRNA levels in small intestine from CONV-R mice treated for 7 days with an antibiotic cocktail (ABX) (n = 6–8 Swiss Webster mice per group). **e–h**, Relative TLR2, 1, 6 and 4 mRNA levels in small intestine from MyD88^−/−^ mice compared to wildtype (WT) controls (n = 6–7 C57BL/6J mice per group). **i–l**, Relative TLR2, 1, 6 and 4 mRNA levels in small intestine from TRIF^−/−^ mice compared to WT controls (n = 6–7 mice per group). **m–o**, Relative TLR1, 6 and 4 mRNA levels in small intestine from TLR2^−/−^ mice compared to WT controls (n = 6–7 mice per group). **p**, Relative TLR2 mRNA levels in small intestine from TLR4^−/−^ mice compared to WT controls (n = 6–7 mice per group). Female mice were analyzed. Results are shown as means ± s.e.m. One asterisk, P<0.05; two asterisks, P<0.01; three asterisks, P<0.005; four asterisks, P<0.001.

Since TLR2 downstream signaling is primarily mediated via the adapter molecule MyD88 [9] whereas the adapter TRIF is required for transduction of signals from TLR3 and TLR4, we next assessed the role of these adapter molecules for the induction of the TLR2 signaling complex in the distal small intestine. Interestingly, MyD88 mRNA levels were increased in CONV-R mice compared with GF controls (**Fig. 2a**), whereas transcript levels of TRIF were decreased by the presence of a gut microbiota (**Fig. 2b**). This could potentially explain hyporesponsiveness of TRIF dependent signaling (e.g. TLR3 and TLR4) in response to microbial colonization. In contrast, 7 day antibiotic treatment of CONV-R mice did not change TRIF transcript levels but decreased MyD88 transcripts (**Fig. 2c,d**). Similar to the situation in GF mice we found reduced TLR2 and TLR1 transcript levels in mice deficient in MyD88 (**Fig. 2e,f**) and again TLR6 and TLR4 transcripts were unchanged (**Fig. 2g,h**). Moreover, TRIF-deficiency likewise resulted in a suppression of TLR2, TLR1, TLR6 and TLR4 mRNA levels (**Fig. 2i–l**) suggesting a cross-talk between TRIF and MyD88 dependent TLR signaling pathways in the small intestine. Interestingly, we found decreased mRNA levels of the TLR2 co-receptors TLR1 and increased levels of TLR6 in Tlr2^−/−^ mice (**Fig. 2m–n**). A cross-talk between TRIF and MyD88-dependent TLR receptors was further inferred by increased TLR4 transcript levels in small intestinal tissues from Tlr2^−/−^ mice (**Fig. 2o**) and increased TLR2 transcripts in small intestinal tissues of Tlr4^−/−^ mice (**Fig. 2p**). This suggests that defective TLR2 sensing could be compensated by increased TLR4 expression and that defective TLR4 sensing may require increased TLR2 expression to maintain intestinal homeostasis.

**Figure 3:**
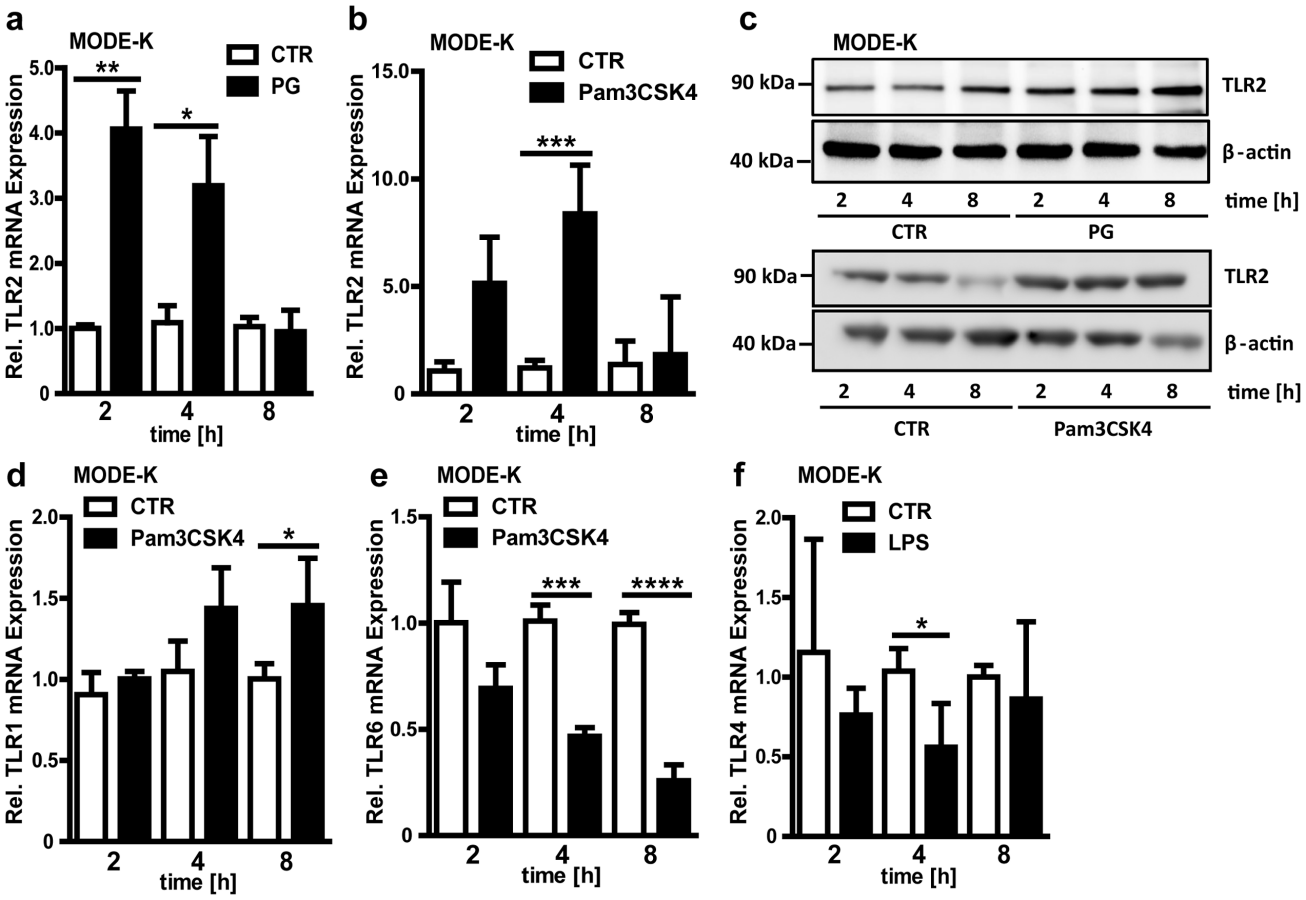
Agonist-specific orchestration of the TLR expression profile in a mouse small intestinal epithelial cell line. **a+b,** Relative mRNA expression of TLR2 in MODE-K cells stimulated with PG (50 µg/ml) or Pam3CSK4 (0.5 µg/ml) for 2, 4 or, 8 hours (n = 4). **c,** TLR2 immunoblot of PG or Pam3CSK4 stimulated MODE-K cells. Cells were treated with or without (CTR) PG or Pam3CSK4 for 2, 4, or 8 hours (n = 3, representative blot). **d–f,** Relative TLR1, 6 and 4 mRNA expression in MODE-K cells stimulated with Pam3CSK4 or LPS for 2, 4, or 8 hours (n = 4). Results are shown as means ± s.e.m. One asterisk, P<0.05; two asterisks, P<0.01; three asterisks, P<0.005; four asterisks, P<0.001.

To further pinpoint the underlying cellular mechanism of these microbial effects on TLR2 and TLR4 expression we used a sterile infection cell culture model. The small intestinal epithelial cell line MODE-K [19] was stimulated for 2, 4 and 8 hours with the bacterial TLR2-agonists peptidoglycan (PG), the synthetic lipoprotein TLR2-agonist Pam3CSK4 and the TLR4-agonist lipopolysaccharide (LPS). TLR2 transcript levels were strongly increased 2 and 4 hours after PG challenge (**Fig. 3a** **)** and TLR2 transcripts were also enhanced after Pam3CSK4 stimulation (**Fig. 3b** **)**. Interestingly, these effects were blunted 8 hours after stimulation with PAMPs. This robust increase in TLR2 expression was also found on the protein level when MODE-K cells were stimulated with PG or Pam3CSK4 (**Fig. 3c**). Transcripts of the TLR2 co-receptor TLR1 were only slightly up-regulated upon longer stimulation periods with the TLR2 agonist Pam3CSK4 (**Fig. 3d**), while TLR6 expression was diminished at longer stimulation periods (**Fig. 3e**). In contrast to TLR4 expression in the small intestine of colonized mice (**Fig. 1e**) and to antibiotic decimation of the gut microbiota (**Fig. 1j**), treatment with LPS for 4h resulted in decreased TLR4 mRNA levels in MODE-K cells (**Fig. 3f**). Collectively, these results demonstrate an agonist-specific orchestration of the TLR expression profile in small intestinal epithelial cells.

**Figure 4:**
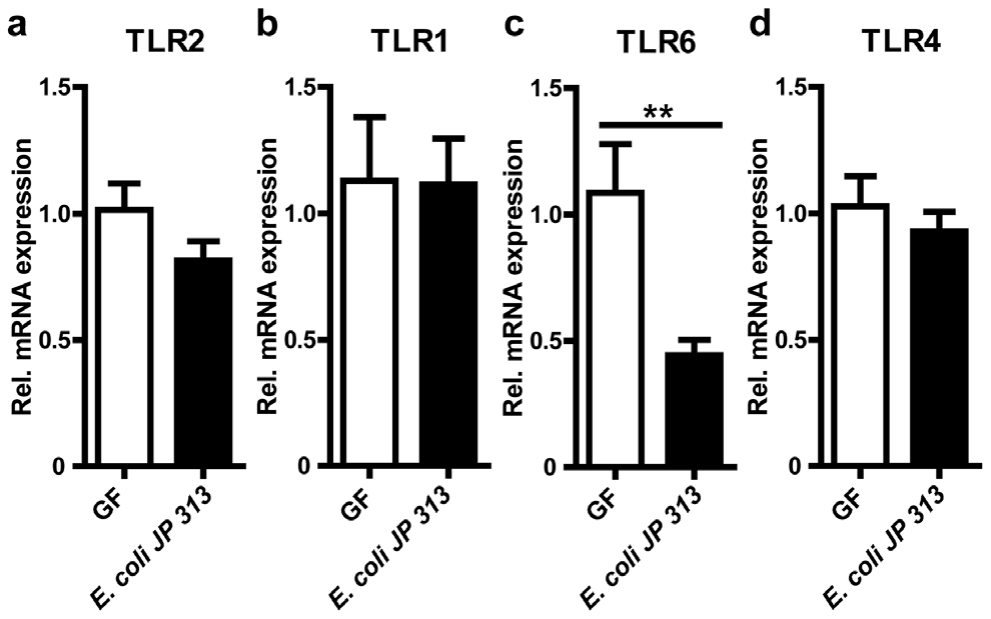
Monocolonization with *E. coli* JP313 decreases TLR6 transcript levels. **a–d,** Relative TLR2, 1, 6, and 4 mRNA levels in small intestine from mice colonized for 14 days with *E. coli* JP313 (n = 7 male C57BL/6 mice per group). Results are shown as means ± s.e.m. Two asterisks, P<0.01.

Since cell culture experiments with *Clostridium butyricum*, a widely used probiotic bacterium, have recently demonstrated the induction of TLR2 expression in the human colon epithelial cell line HT-29 [20], we decided to further explore the effects of an individual microbial colonizer on the orchestration of the TLR2 signaling complex by the use of GF mouse isolator technology. To this end, we colonized GF C57BL/6 mice with the *E. coli* strain JP 313 for a time period of 14 days. Monocolonization with the *E. coli* strain JP 313 did not alter mRNA levels of TLR2, TLR1 and TLR4 (**Fig. 4a,b,d**), but significantly reduced transcript levels of the TLR2 co-receptor TLR6 (**Fig. 4c**). This implies that colonization with single microbes could specifically affect expression of epithelial pattern recognition receptors.

**Figure 5:**
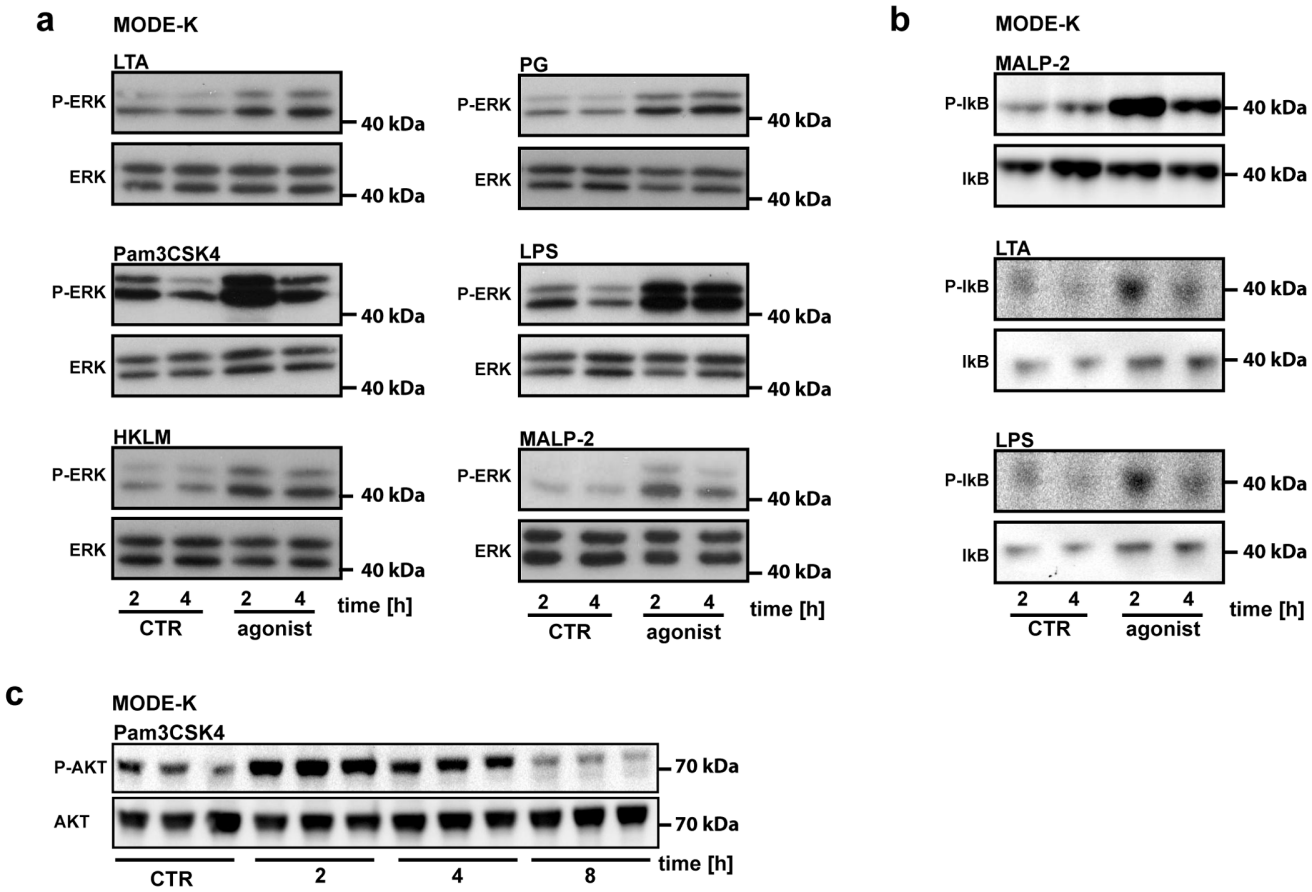
MODE-K cells are reactive to TLR2 activating PAMPs and LPS. **a,** Detection of ERK1/2-phosphorylation of TLR-agonist (LTA, 10 µg/ml; Pam3CSK4, 0.5 µg/ml; HKLM, 2×10^5^ cells per well; PG, 50 µg/ml; MALP-2, 2 µg/ml; LPS, 100 ng/ml) treated MODE-K cells (n = 4–10). **b,** Detection of IkB-phosphorylation of TLR-agonist treated MODE-K cells (n = 3). **c,** Detection of AKT-phosphorylation of Pam3CSK4 treated MODE-K cells (n = 3).One representative immunoblot is shown for each agonist and kinase.

Since we found that the gut microbiota and even individual colonizers impact on epithelial TLR2/1 or TLR2/6 receptor component expression, we next analyzed if relevant downstream kinase signaling pathways could be activated in epithelial cells by stimulation with PAMPs. In the sterile infection MODE-K model, stimulation with the TLR2 agonists lipoteichoic acid (LTA), Pam3CSK4 and heat-killed *Listeria monocytogenes* (HKLM) readily activated TLR downstream signaling pathways as shown by phosphorylation of ERK1/2 (mitogen-activated protein kinase 1 and 2) (**Fig. 5a**). ERK phosphorylation was also increased by stimulation with PG, LPS and MALP-2. The bacterial TLR2-agonists Macrophage-activating lipopeptide-2 (MALP-2), LTA and LPS-induced phosphorylation of the inhibitor of Nuclear Factor kappa-light-chain-enhancer of activated B cells (IκB), the natural inhibitor of the transcription factor NFκB (**Fig. 5b**). Furthermore, Protein-kinase B (AKT)-phosphorylation could also be triggered by stimulation with Pam3CSK4 in the MODE-K sterile infection model (**Fig. 5c**). Comparable to the changes in TLR2 expression (**Fig. 3a**), the increase in phosphorylation was most intense 2 to 4 hours upon stimulation (**Fig. 5a–c**) and decreased to levels of unstimulated controls at 8 hours upon stimulation (**Fig. 5c**). Collectively, we show that the small intestinal epithelial cell line MODE-K is reactive to TLR2 activating PAMPs via several protein kinase pathways related to cellular proliferation.

**Figure 6:**
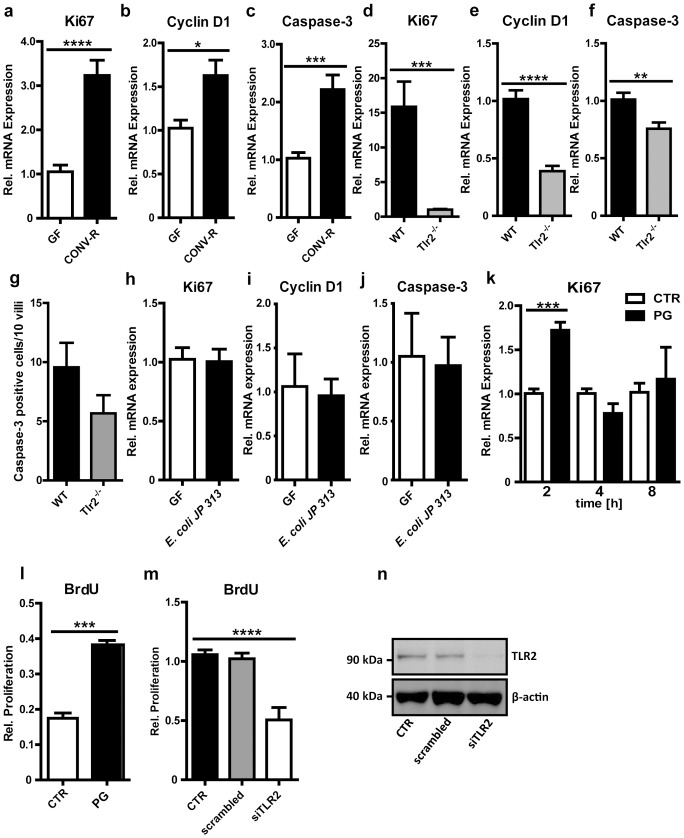
TLR2 signals in the small intestinal epithelium evoke a proliferation response and increase signs of apoptosis in terminally differentiated epithelium. **a–c**, Relative Ki67, Cyclin D1 or Caspase-3 mRNA levels in small intestine from GF and CONV-R mice (n = 7 Swiss Webster mice per group). **d–f**, Relative Ki67, Cyclin D1 or Caspase-3 mRNA levels in small intestine from Tlr2^−/−^ mice compared with WT controls (n = 7 C57BL/6J mice per group). **g**, Number of Caspase-3 positive cells per 10 villi in small intestinal samples from WT or Tlr2^−/−^ mice. **h–j**, Relative Ki67, Cyclin D1 and Caspase-3 mRNA levels in small intestine from mice colonized for 14 days with *E. coli* JP313 (n = 7 C57BL/6 mice per group). **k**, Relative Ki67 mRNA levels in MODE-K cells stimulated with PG (50 µg/ml) for 2, 4, or 8 hours (n = 4). **l**, Relative proliferation of PG treated MODE-K cells measured by incorporation of BrdU compared to untreated control cells (n = 3). **m**, Relative proliferation of MODE-K cells transfected with siRNA against TLR2 or scrambled control RNA measured by incorporation of BrdU (n = 4). **n**, TLR2 immunoblot of siRNA transfected MODE-K cells. Results are shown as means ±s.e.m. One asterisk, P<0.05; two asterisks, P<0.01; three asterisks, P<0.005; four asterisks, P<0.001.

A pivotal role of MyD88-dependent pattern recognition has previously been suggested for proliferation and differentiation of the gut epithelial lining [21] and a mitogenic effect has been demonstrated for TLR2 agonists in a human lung epithelial cell line [22]. However, the induction of the proliferative cell response in intestinal epithelial cells has not been unambiguously assigned to TLR2 in previous studies. The presence of an intestinal microbiota results in increased renewal of the epithelial lineage [3], [6], [8] as indicated by increased mRNA levels of the proliferation marker Ki67 in CONV-R mice compared with GF controls (**Fig. 6a**). Increased small intestinal cell turn over was further corroborated by increased Cyclin D1 mRNA levels (**Fig. 6b**), which drives G1/S phase transition, and increased mRNA levels of the apoptotic marker Caspase-3 (**Fig. 6c**). Since TLR2 is up-regulated upon microbial colonization, we reasoned that TLR2 signaling could stimulate the proliferation of small intestinal epithelial cells. Indeed, transcript levels of the proliferation marker Ki67 and Cyclin D1 were vastly reduced in Tlr2^−/−^ mice (**Fig. 6d,e**). In support of decreased cell turn over in Tlr2^−/−^ mice, mRNA levels of the apoptotic marker Caspase-3 were also decreased (**Fig. 6f**). Reduced apoptosis in the small intestine of Tlr2^−/−^ mice was accompanied by decreased numbers of cleaved Caspase-3 positive cells in the villus structures (**Fig. 6g** and **Fig. S1**). In addition to TLR2 deficiency, the markers of cell proliferation (Ki67), cell turn over (Cyclin D1) and apoptosis (Caspase-3) were also changed in the ileum of Tlr4^−/−^ mice, but not in Tlr5^−/−^ mice, indicating that intestinal homeostasis could be influenced by diverse PAMPs (**Fig. S2**). Only Caspase-3 was affected in the Tlr7^−/−^ mice, whereas Ki67 and Cyclin D1 were unchanged (**Fig. S2**). The role of TLR signaling on the expression of apoptotic markers was in line with the significant increase in TUNEL-positive nuclei in small intestinal villus structures of CONV-R mice compared with GF controls that are devoid of gut microbial TLR activation (**Fig. S3a,b**). Interestingly, monocolonization with the *E. coli* strain JP 313 was not sufficient to alter small intestinal cell turn over as Ki67, Cyclin D1 and Caspase-3 transcript levels were unchanged (**Fig. 6h–j**). In accordance with decreased small intestinal tissue renewal observed in GF and Tlr2^−/−^ mice, we found that 2 h stimulation of MODE-K cells with the TLR2 agonist PG resulted in a pronounced proliferation response as indicated by elevated Ki67 transcript levels (**Fig. 6k**). In line, BrdU incorporation of MODE-K cells was increased by 2-fold after 24 hours of PG stimulation (**Fig. 6l**). We could demonstrate that pattern recognition induced cell proliferation was clearly TLR2 mediated since siRNA silencing of TLR2 expression led to a vast reduction of BrdU incorporation of PG stimulated MODE-K cells (**Fig. 6m,n**). Our results indicate that the orchestration of TLR2 signals in terminally differentiated small intestinal epithelial cells regulates cell turn over by evoking a pronounced epithelial proliferation response that is accompanied by a modest increase in apoptosis.

## Discussion

Here, we report that expression of the epithelial pattern recognition receptor TLR2 and its co-receptor TLR1 in the small intestinal mucosa is tuned by PAMPs of the intestinal microbiota (**Fig. 1**). Our results with Myd88^−/−^ and Trif^−/−^ mouse lines suggest that transcriptional regulation of TLRs in the small intestine depends on the adapter molecules MyD88 and TRIF, which also underlay a regulation by the gut microbiota (**Fig. 2**). Microbiota-driven TLR expression is a reversible process as antibiotic decimation of gut microbial communities led to reduced transcript levels of various TLRs (e.g. TLR1, TLR2, TLR6 and TLR4) (**Fig. 1**). Both Myd88^−/−^ and Trif^−/−^ impaired expression of TLR2/1 and TLR2/6 in the ileum (**Fig. 2e, g** and **Fig. 2i–k**). Interestingly, Tlr2-deficiency leads to decreased TLR1 transcripts, but increases TLR6 transcript levels (**Fig. 2m,n**). Furthermore, we found that Tlr2-deficiency impacts on ileal TLR4 mRNA levels and vice versa (**Fig. 2o,p**). In accordance with a previous cell culture study [20], we could specifically provoke alterations in TLR transcript levels in the small intestine by monocolonization with *E. coli* JP 313 (**Fig. 4**). Up-regulation of the TLR2 signaling complex by TLR2 agonists could be confirmed in a cell culture model using the small intestinal epithelial cell line MODE-K (**Fig. 3a–f**). Interestingly, this terminally differentiated intestinal epithelial cell line is able to recognize PAMPs. Pam3CSK4 signals via the TLR2/1 complex, while PG triggers diverse signaling pathways [23]. Increased TLR2 transcripts upon Pam3CSK4 stimulation (**Fig. 3b**) along with modestly increased TLR1 transcripts (**Fig. 3d**) and decreased TLR6 transcripts (**Fig. 3e**) suggest that stimulation with the TLR2/1 agonist Pam3CSK4 augments signaling though its utilized signaling complex TLR2/1, but impairs TLR2/6 signaling. Expression profiling of cell cycle and proliferation markers in small intestinal tissues from GF and CONV-R mice and from Tlr2^−/−^ mice demonstrates a role for TLR2 in small intestinal cell turn over (**Fig. 6a–g**), but this proliferation response could not be evoked by monocolonization with the *E. coli* strain JP 313 (**Fig. 6h–j**). With a sterile infection cell culture model and siRNA silencing of TLR2 we were able to specifically pinpoint the induction of the epithelial proliferation response to TLR2 (**Fig. 6k–n**). These findings are further corroborated by cell culture experiments which demonstrated a role for TLR2 in activation of diverse protein kinase pathways that are involved in epithelial proliferation of terminally differentiated small intestinal epithelial cells (**Fig. 5**).

Although it is evident that innate immune sensing augments renewal of the intestinal epithelial lining primarily from the stem cell niche situated at the base of the crypts of Lieberkühn [1], [8], we suggest that the TLR2 dependent proliferation response that can be evoked in differentiated small intestinal epithelial cells (MODE-K) might represent an additional route that ensures integrity of the intestinal lining during renewal of the intestinal mucosa (**Fig. S4**). This proliferative adaption to microbial stimuli could serve to support efficient nutrient uptake in the distal small intestine. A recent study with GF rats has demonstrated a role for the gut microbiota and for *E. coli* in the augmentation of colonic epithelial cell proliferation as indicated by elevated proliferation markers, but the microbial components that unleash this proliferation response and the activated signaling pathways were not delineated [24], [25]. Our data suggest that increased epithelial cell proliferation (**Fig. 6**) could be due to increased TLR2 signaling resulting in activation of the ERK1/2 and AKT pathways (**Fig. 5**). These results are in line with previous cell culture studies demonstrating the dependence of intestinal epithelial cell proliferation on ERK1/2 and AKT signaling [26], [27]. Further studies are needed to identify which cell cycle stimulatory factors are specifically induced via TLR2 on small intestinal epithelial cells that can act in an autocrine or paracrine manner to induce proliferative and apoptotic pathways in the small intestinal mucosa and to pinpoint the possible role of ROS signaling pathways that are linked to innate immune signaling and renewal of the intestinal epithelium [28].

Interestingly, decimation of the gut microbiota by treatment with broad-spectrum antibiotics diminished transcript levels of various TLRs in the small intestine of CONV-R mice (**Fig. 1f–j**). This finding implies that eradication of colonizing microbial communities during antibiotic therapy may possibly attenuate epithelial innate immune sensing and hence to some extent limit the development of inflammation. This in some cases might be a double edged sword, since antibiotic treatment may select for resistant germs and at the same time disable protective innate immune responses. Furthermore, there is mounting evidence for that deficiency in pattern recognition could select for colonization with pathobionts that may exert deleterious effects in the absence of an adequate inflammatory response [29]. Our data on small intestinal TLR2 expression imply a crosstalk between MyD88 and TRIF-dependent TLR signaling circuits [30], [31], since TLR2 expression is affected in both MyD88 and TRIF-deficient mouse lines (**Fig. 2e–l**). Further, we found that deficient TLR4 signaling augments TLR2 transcripts and vice versa (**Fig. 2m–p**). Since it is not sufficiently resolved how stimulation of TLRs with specific agonists or genetic deficiency of individual TLRs affects the expression of other receptors of this family, the context-specific role of the crosstalk between MyD88 and TRIF-dependent signaling pathways in the modulation of TLR expression in the small intestinal epithelium will deserve further investigation. Such efforts will be inevitable to understand the complexity of TLR signaling and its role in small intestinal tissue homeostasis.

While a robust effect of a complex gut microbiota was observed on small intestinal TLR2 expression (**Fig. 1a–c**), renewal and cell turn over (**Fig. 6a–c**), it was not possible to augment mucosal proliferation in the small intestine by colonization with the proteobacterial colonizer *E. coli* JP 313 (**Fig. 6h–j**) [32]. This strain of *E. coli* inoculated alone did not trigger mucosal proliferation possibly due to the fact that colonization with JP 313 alone is not sufficient to counterbalance mucosal atrophy observed in GF mice [5], [24]. The situation in monocolonized mice is in contrast to cell culture experiments with PG stimulated MODE-K cells where a pronounced TLR2-dependent proliferation response could be evoked (**Fig. 6k–n**). Hence, monocolonizations with additional gut microbes and importantly with bacterial deletion mutants that target PAMP synthesis will be mandatory to reveal the significance of individual microbes and bacterial membrane components for TLR induction and epithelial cell proliferation.

In addition to the identified role of TLR2 in gut epithelial cell proliferation this pattern recognition receptor has recently been implicated in permeability regulation of the epithelial lining [33]. The regulation of barrier function is one way how TLR2 could affect host metabolism, but there is increasing evidence for additional modes of action [34]. TLR2 has been shown to have profound effects on the composition of gut microbial communities [34], [35]. A recent study suggests that alterations in gut microbiota composition as they are found in TLR deficient mice may rather be caused by familiar transmission than by innate deficiency [36]. Alterations in the diversity of the gut microbiota are well known to impact on host metabolism [34]. These recent reports clearly point to roles of TLR2 beyond regulation of small intestinal tissue homeostasis. In this context, further experiments including cell-specific targeting of TLR2 are required to differentiate the effects arising from the myeloid lineage from the effects that are originating from epithelial cells.

Previous work has established the protective role of TLR2 and TLR4 and of the gut microbiota to resist to chemical induced epithelial injury [21]. Mice that were deficient in MyD88-dependent signaling or mice subjected to antibiotic treatment resulting in deciminated indigeneous microbiota showed a vastly increased lethality during long term DSS treatment. A defect of steady-state intestinal epithelial homeostasis in absence of TLR signaling was suggested to be fundamental for the increased susceptibility to epithelial damage [21]. Indeed, the induction of a proliferation response in terminally differentiated intestinal epithelial cells via TLR2 could be beneficial under conditions where enhanced renewal and mucosal repair is required, e.g. in radiotherapy or chemotherapy-induced intestinal mucositis [37]. With respect to these relevant clinical complications further studies are needed to pinpoint the exact role of innate immune signaling in mucosal repair processes.

## Supporting Information

Figure S1 **TLR2-deficiency leads to decreased signs of apoptosis in the small intestine.** Stainings of Caspase-3 expression in small intestinal tissue sections from WT and Tlr2^−/−^ mice. Sections were embedded in paraffin, cut in 8 µm sections and stained with an anti-Caspase-3 antibody. 20x magnifications are shown.
(TIF)

Figure S2 **Cell turn over is changed in the ileum of Tlr4^−/−^ and Tlr7^−/−^ mice, but not in Tlr5^−/−^ mice.** **a–c,** Relative Ki67, Cyclin D1 and Caspase-3 mRNA levels in small intestine from Tlr4^−/−^ mice compared with WT controls (n = 7 female mice per group). **d–f,** Relative Ki67, Cyclin D1 and Caspase-3 mRNA levels in small intestine from Tlr5^−/−^ mice compared with WT controls (n = 6 male mice per group). **g–i,** Relative Ki67, Cyclin D1 and Caspase-3 mRNA levels in small intestine from Tlr7^−/−^ mice compared with WT controls (n = 5–8 mice per group). Results are shown as means ± s.e.m. One asterisk, P<0.05; two asterisks, P<0.01; four asterisks, P<0.001.
(TIF)

Figure S3 **The gut microbiota increases apoptosis in the small intestine.** **a,** TUNEL-staining of small intestinal tissue sections of GF and CONV-R female Swiss Webster mice. Paraffin-embedded samples were cut in 8 µm sections. Tissues were deparaffinized, rehydrated and nicks were FITC labeled by the Terminal deoxynucleotidyl Transferase (TdT) reaction. Apoptotic cells are stained with fluorescein (green), nuclei are stained with DAPI (blue). 20x magnifications are shown. **b,** Quantitative analysis of TUNEL-positive cells in GF and CONV-R tissue sections. Results are shown as means ± s.e.m. Four asterisks, P<0.001.
(TIF)

Figure S4 **Model delineating the role of the gut microbiota on TLR2 agonist stimulated increase in downstream kinase signaling, TLR2 expression and cell turn over of terminally differentiated enterocytes in the ileum.**
(TIF)
