# Supplementary figures and images for: Gut Microbial Colonization Orchestrates TLR2 Expression, Signaling and Epithelial Proliferation in the Small Intestinal Mucosa
Source: PLoS One. 2014 Nov 14;9(11):e113080. doi: 10.1371/journal.pone.0113080 (PMC4232598; doi:10.1371/journal.pone.0113080)

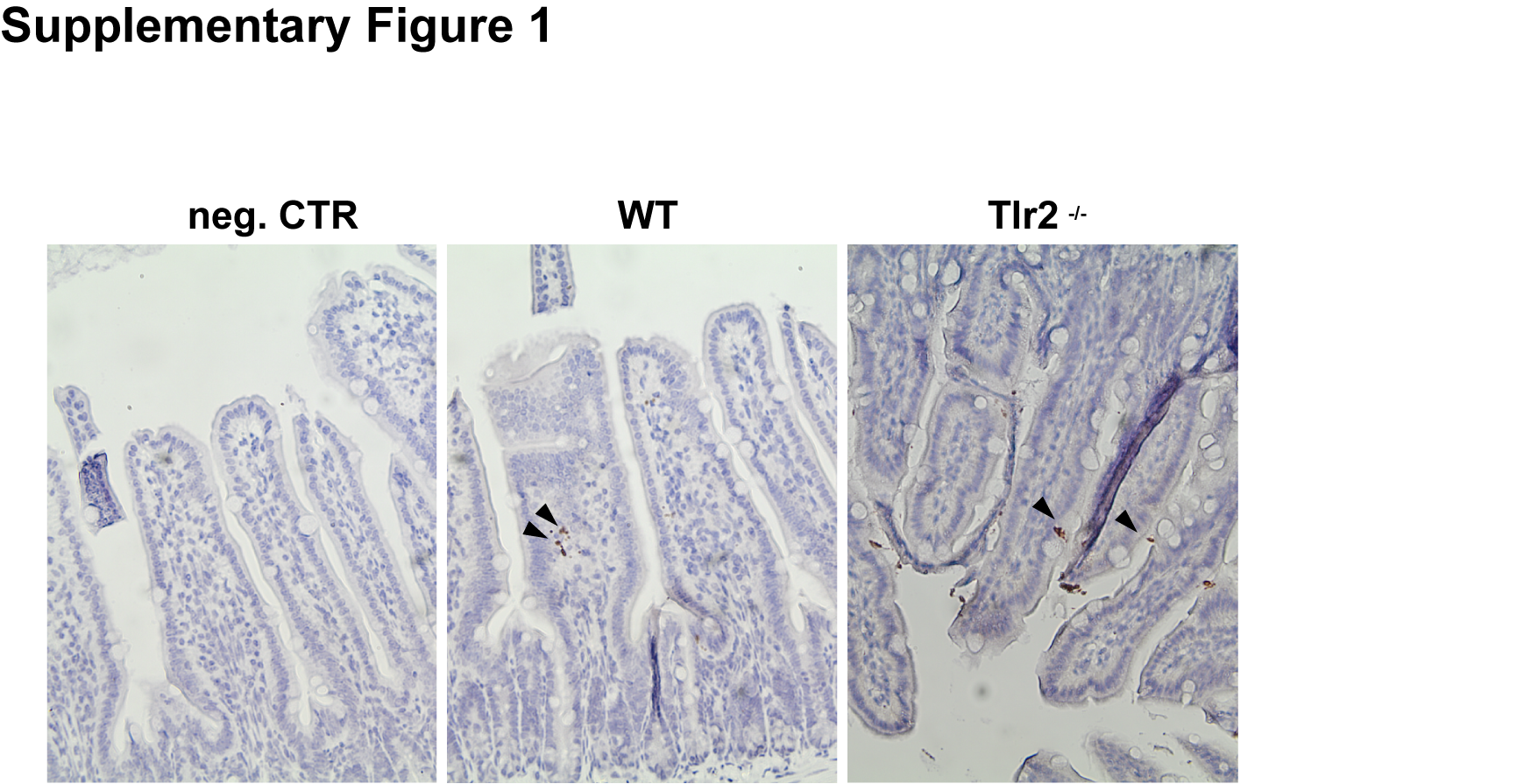

Supplement: Figure S1 — TLR2-deficiency leads to decreased signs of apoptosis in the small intestine. Stainings of Caspase-3 expression in small intestinal tissue sections from WT and Tlr2−/− mice. Sections were embedded in paraffin, cut in 8 µm sections and stained with an anti-Caspase-3 antibody. 20x magnifications are shown. (TIF) [file pone.0113080.s001.tif]

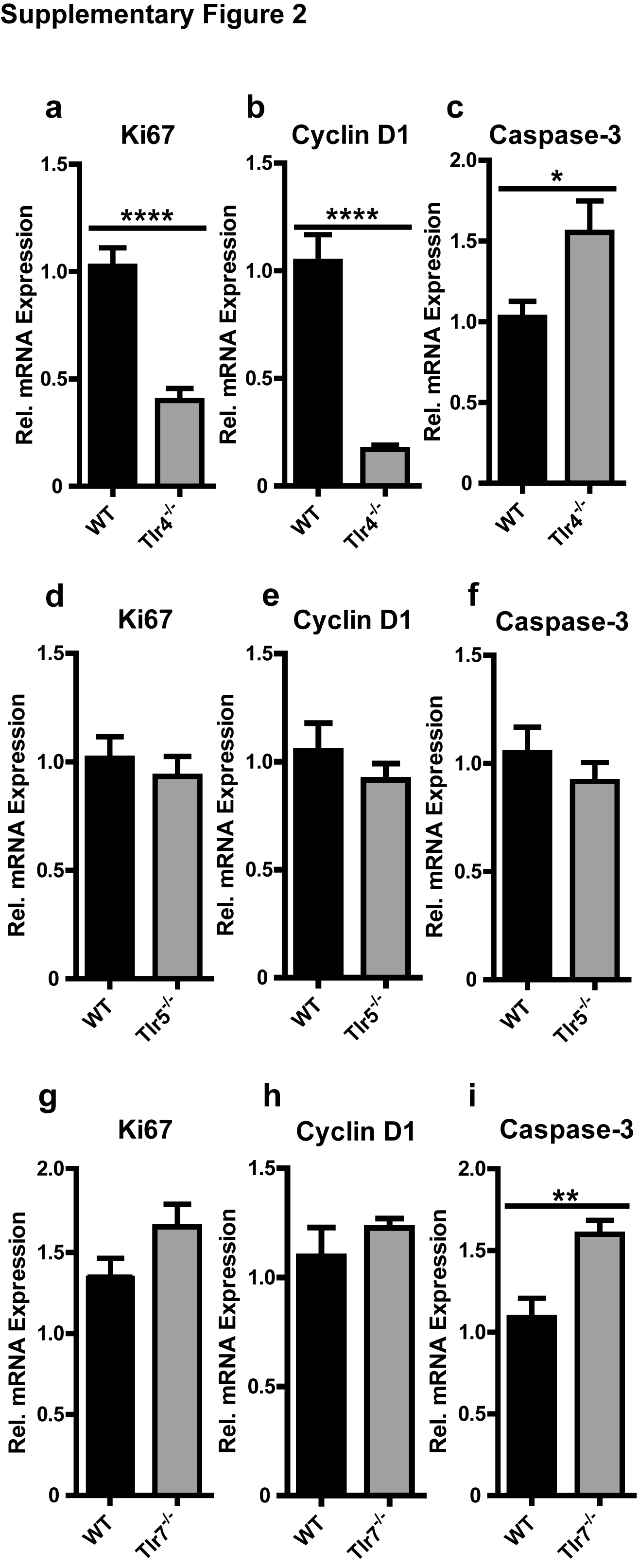

Supplement: Figure S2 — Cell turn over is changed in the ileum of Tlr4−/− and Tlr7−/− mice, but not in Tlr5−/− mice. a–c, Relative Ki67, Cyclin D1 and Caspase-3 mRNA levels in small intestine from Tlr4−/− mice compared with WT controls (n = 7 female mice per group). d–f, Relative Ki67, Cyclin D1 and Caspase-3 mRNA levels in small intestine from Tlr5−/− mice compared with WT controls (n = 6 male mice per group). g–i, Relative Ki67, Cyclin D1 and Caspase-3 mRNA levels in small intestine from Tlr7−/− mice compared with WT controls (n = 5–8 mice per group). Results are shown as means ± s.e.m. One asterisk, P<0.05; two asterisks, P<0.01; four asterisks, P<0.001. (TIF) [file pone.0113080.s002.tif]

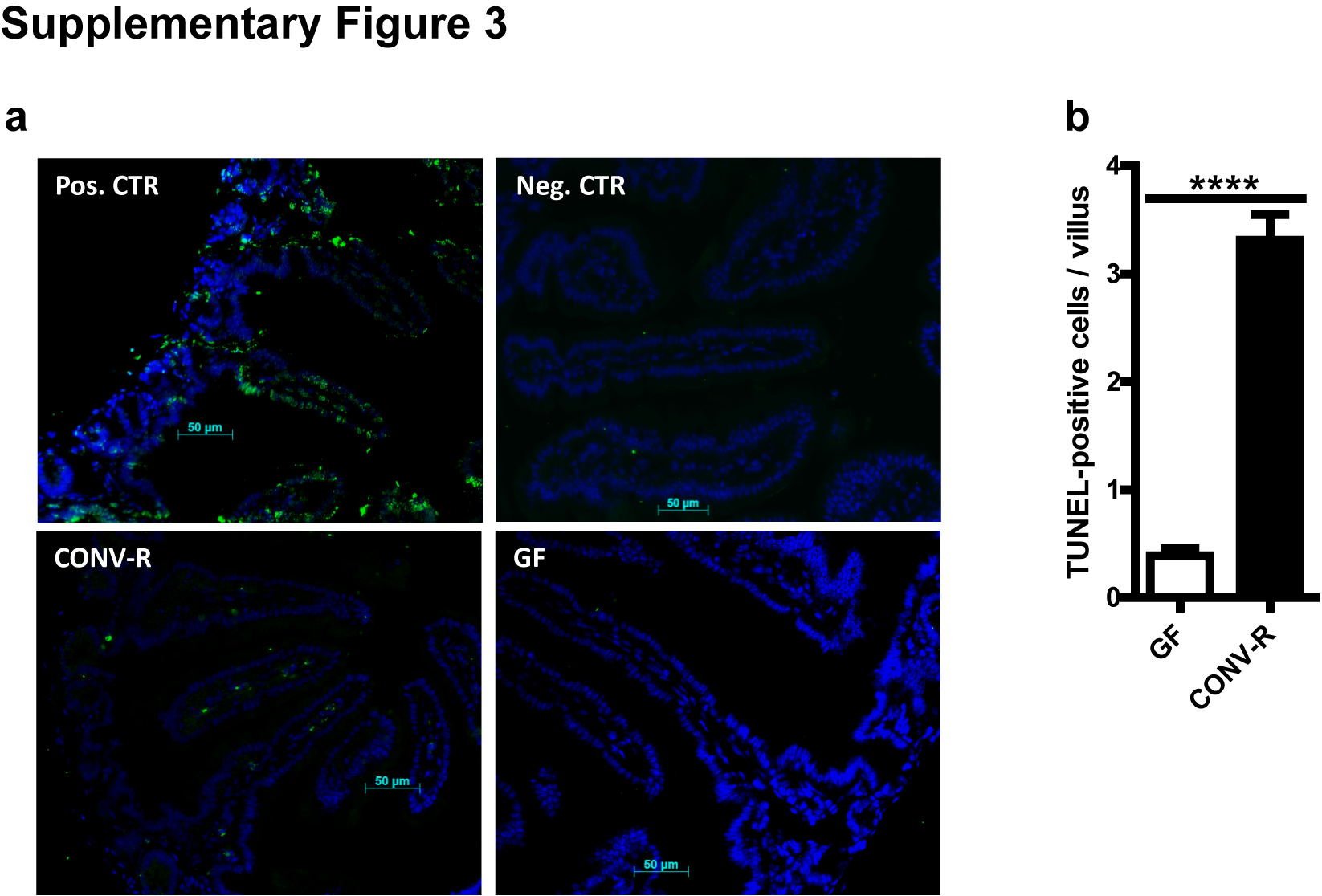

Supplement: Figure S3 — The gut microbiota increases apoptosis in the small intestine. a, TUNEL-staining of small intestinal tissue sections of GF and CONV-R female Swiss Webster mice. Paraffin-embedded samples were cut in 8 µm sections. Tissues were deparaffinized, rehydrated and nicks were FITC labeled by the Terminal deoxynucleotidyl Transferase (TdT) reaction. Apoptotic cells are stained with fluorescein (green), nuclei are stained with DAPI (blue). 20x magnifications are shown. b, Quantitative analysis of TUNEL-positive cells in GF and CONV-R tissue sections. Results are shown as means ± s.e.m. Four asterisks, P<0.001. (TIF) [file pone.0113080.s003.tif]

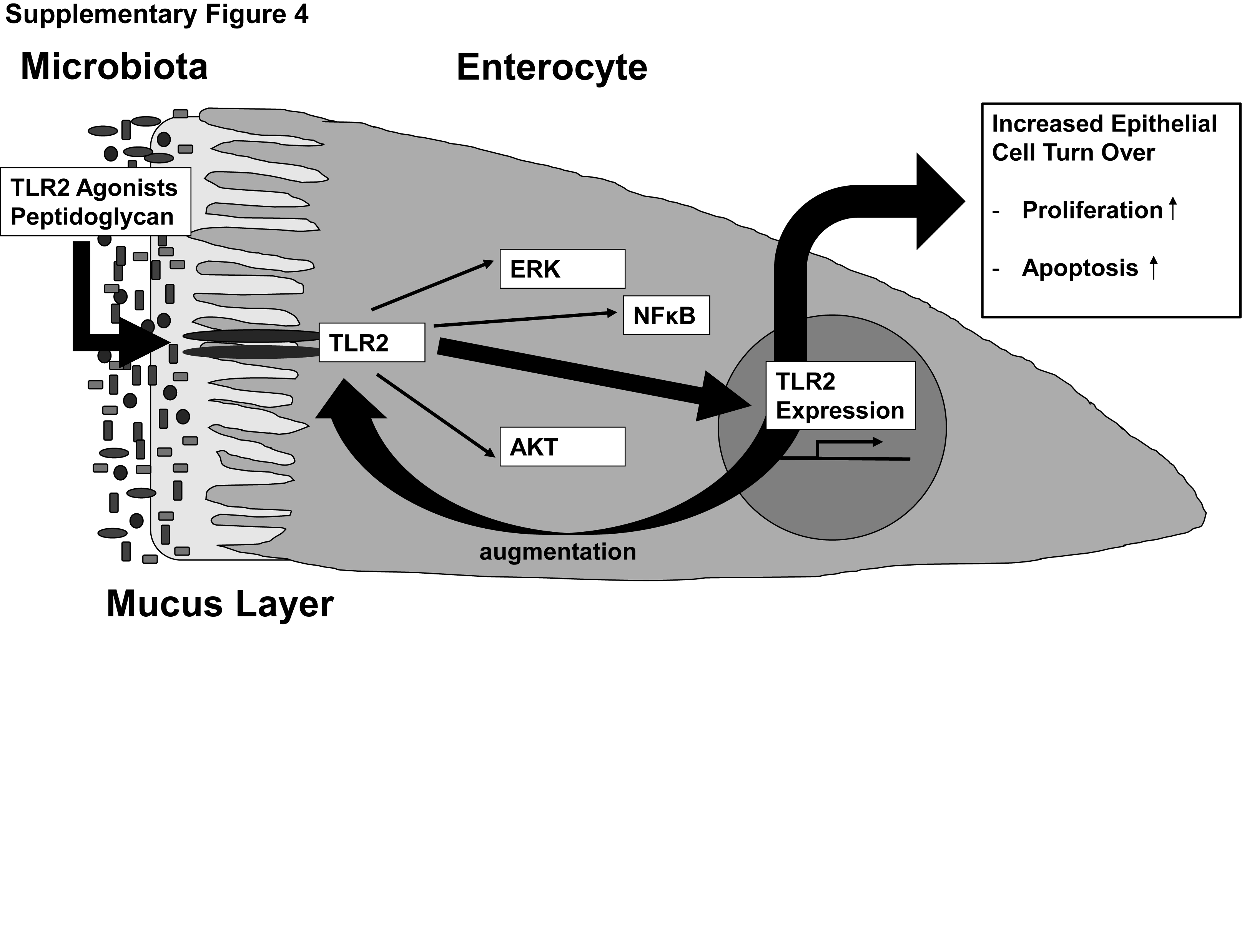

Supplement: Figure S4 — Model delineating the role of the gut microbiota on TLR2 agonist stimulated increase in downstream kinase signaling, TLR2 expression and cell turn over of terminally differentiated enterocytes in the ileum. (TIF) [file pone.0113080.s004.tif]
